# 

**Published:** 1863-01

**Authors:** 


### INDEX.

| | Page. |
|---|---|
| Arsenic and its compounds, | 9 |
| Address, by J. Mansfield, | 129 |
| A Lusus Naturæ, by E. Collins, | 134 |
| Annual Commencement of the "Penn. College of Dental Surgery," | 158 |
| Artificial Dentures, by W. H. Atkinson, M. D., | 165 |
| "An Experiment" examined, by Dr. B. Wood, | 203 |
| Anæsthetica, | 218 |
| Amalgam Fillings, by G. F. Foote, M. D., | 241 |
| Amalgam, | 264 |
| An extension thimble, | 277 |
| An Address, by Dr. S. B. Palmer, | 285 |
| Alveolar abscess, | 299 |
| Animal life, | 304 |
| American Dental Convention, | 314 |
| American " " | 318 |
| American Dental Association, | 318 |
| American Hard Rubber Co. against J. T. Toland, | 319 |
| Abnormal development of the teeth, | 328 |
| Air Chambers, by J. D. Anderson, | 337 |
| American Dental Convention, | 364 |
| A magnificent hospital, | 364 |
| American Dental Association, | 365 |
| A Report on the Condition and Organization of Local Societies, by J. Taft, | 369 |
| Another death from chloroform, | 406 |
| A sensible arrangement, | 416 |
| A case, | 416 |
| Anomalous cases, by W. H. Atkinson, | 430 |
| Absorptive powers of the skin, | 448 |
| Anæsthetics, by J. D. White, D. D. S., | 465 |
| Absorption of Fangs of the Temporary Teeth, by W. H. Atkinson, M.D., | 473 |
| | |
| Bibliographical, | 236 |
| Back numbers, | 368 |
| Bibliographical, | 548 |
| | |
| Correspondence, | 83 |
| Cincinnati Dental Association, | 98 |

Correspondence, .......................................................................... 134
Case of hemorrhage from a branch of the anterior palatine artery, by H. T. Kempton, .......................................................... 141
Correspondence,.......................................................................... 158
Concerning the opening of abscesses, by Nullopath,........................... 186
Cincinnati Dental Association,.......................................................... 192
Correspondence,.......................................................................... 215
Correspondence,.......................................................................... 247
Case No. 11 and 12, ...................................................................... 316
Causes retarding dental progress, by W. H. Atkinson,......................... 321
Case of fracture of inferior maxilla, treated by A. Gripe,.................... 362
Case of successful removal of foreign body in the throat, by tracheotomy, ................................................................................ 407
Care of the eyes in the use of the microscope,.................................... 411
Causes retarding Dental Progress, by W. H, Atkinson, ......................... 321
Correspondence,.......................................................................... 435
Crust of bread, ............................................................................ 493
Chloride of iron and chloride of sodium as a hæmostatic,..................... 494
Carbolic acid,.............................................................................. 539
Close of the year,......................................................................... 545

Dentition and its derangements,....................................................... 36
Does breathing through the mouth injure the teeth? by George F. Foote, M. D.,.............................................................................. 60
Dentition and its derangements, by A. Jacobi, M. D.,........................... 86
District Court—Judge Hare—the use of chloroform,............................... 95
Dental fees and etiquette, by A. Benedict,.......................................... 114
District Court—Judge Hare—the use of chloroform,............................... 121
Dentition and its derangements, by A. Jacobi, M. D.,........................... 135
Dental materials,.......................................................................... 146
Dentition and its derangements, by A. Jacobi, M. D.,........................... 160
Developments of dental tissues, by W. H. A.,...................................... 237
Disinfectants and their application to Therapeutics,............................ 269
Development of dental tissues, by W. H. A., ...................................... 279
Dental Physiology, by W. H. Atkinson, M. D.,..................................... 384
Dental matters in Iowa,.................................................................. 435
Dangerous forms of quackery,.......................................................... 446
Diathesis, .................................................................................. 454
Dental Colleges,........................................................................... 460
Does the Dental Profession progress?................................................ 516
Deaths from chloroform,................................................................. 535

Editorial,................................................................................... 50
Editorial,................................................................................... 98
Elements of professional succees, by B. P. Aydelott, M.D , D.D,.......... 101
Editorial,................................................................................... 144

Editorial, ........ 189
Editorial, ........ 233
Editorial, ........ 364
Epulis, by Leon. F. Harvey, ........ 417
Editorial, ........ 424
Extirpation of gland, ........ 271
Editorial, ........ 316
Ether as an anæsthetic, ........ 359
Effects of tobacco-smoking, ........ 442
Editorial, ........ 507
Electrical phenonena in the Alps, ........ 495
Editorial, ........ 545

Fang filling, ........ 52
Frankness, honesty, truth,—A Review, by J. D. Anderson, D. D. S., ... 83
Fee bill, ........ 98
Fang filling, by W. H. Shadoan, ........ 399
Fossil man, ........ 404
First annual meeting of the Dental Association of Western New York, 483
Fermentation as a cause of various diseases, ........ 496

Gold-dust and iron-filings as an antidote for corrosive sublimate, ...... 251
Great Western Sanitary Fair, ........ 547

Hudson's Tooth Paste, ........ 51
Hygienic and therapeutic relations of good teeth, ........ 142
Hints on Dental Societies, ........ 414
Hair-lip and cleft palate, ........ 502
Hygienic importance of light, ........ 503
History of Dental Practice on the Pacific Coast, ........ 521
Hæmostatic treatment of cholera, hæmorrhage, exhaustion, etc., ........ 537

Important India Rubber case, ........ 43
Introductory Address, by Anthony Hockley, M. C. D. E., ........ 170
Influence of the sun's rays in the production of organic matter, ........ 261
Institutes of Dental Science, by W. H. Atkinson, M. D., ........ 332
Iodide of potassium and carbonate of potash in rheumatism, ........ 491
Iowa State Dental Society, ........ 507
Iodine, by H. A. Smith, D. D. S., ........ 511

Kalamazoo, Michigan, Dental Society, ........ 80

Leeches again, ........ 50
Leslie's Reguline Gold, ........ 146
Letter from "J." ........ 248
Letter from St. Louis, ........ 250
Lectures on Dental Materia Medica, by Robert T. Hulme, ........ 525

Metals and alloys, by Dr. B. Wood, ........................................ 23
Mistakes of the types,........................................ 51
Mississippi Valley Association,........................................ 53
Metals and alloys, by Dr. B. Wood,........................................ 70
Metals " " " ........................................ 147
Metals " " " ........................................ 193
Meeting of the dentists of Syracuse,........................................ 245
Meeting of Dental Society of Western New York,........................................ 293

Nineteenth Annual Meeting of the Mississippi Valley Association of Dental Surgeons,........................................ 131
Nitrous oxide in asphyxia, by George J. Zeigler, M. D.,........................................ 163
Notes of cases,........................................ 274
Necrosis of lower jaw,........................................ 495
Nerve instruments,........................................ 546

Ohio Dental College Association,........................................ 53
Obituary, ........................................ 99
Ohio College of Dental Surgery,........................................ 190
On the introduction of Specialties into Belvue Hospital,........................................ 227
On the use of glycerine in Surgery and Medicine........................................ 255
Odontographic Society of Pennsylvania,........................................ 319
Obituary, ........................................ 368
On an improved mode of using refrigeration as an anæsthetic and a remedy, ........................................ 451
Obituary, ........................................ 548

Professional etiquette, by W. H. Atkinson, M. D.,........................................ 31
Proximal cavities, by Dr. J. S. Latimer,........................................ 55
Personal, ........................................ 146
Professional individuality, by George F. Foote, M. D.,........................................ 154
Proceedings of the Dental College Association,........................................ 156
Patience and patients,........................................ 189
Practical thoughts, by Dr. W. A. Pease,........................................ 209
Parched-corn meal,........................................ 229
Practical thoughts,........................................ 233
Practical hints,........................................ 259
Plastic fusible metal for filling teeth, by B. Wood, ........................................ 392
Pressure at the bottom of the Atlantic,........................................ 410
Plugging teeth with gold, by B. Wood, M. D.,........................................ 476
Power of the imagination,........................................ 493
Physical effects of exercise upon the human body,........................................ 499
Pittsburg Dental Society,........................................ 509
Preservation of carious teeth by filling, by Enoch C. Rolfe, M. D.,...... 540

"Que va a la Chasse perd sa place,"........................................ 231

Robinson's American Tooth Paste,........................................................ 50
Retrospective and apologetic, by C. C. Dills,........................................ 69
Rubber attachment, ............................................................................ 276
Restoring to its natural appearance a putrefied dead body,.................. 308
Red vulcanite as a base for artificial teeth........................................... 449

Success in the Dental Profession, by C. C. Dills,................................. 16
Success " " " " ........................................ 63
Specialists in Medicine,.......................................................................... 224
Steatomatous cyst upon the tongue,........................................................ 226
Sensible advice,........................................................................................ 232
Scavengers of Nature,.............................................................................. 306
Suggestion,................................................................................................ 315
Something new,........................................................................................ 319
Salivary calcalus,...................................................................................... 407
Sugar as an anthelmintic,........................................................................ 495
Sulphur rendered soft,.............................................................................. 501

The physical knowledge,......................................................................... 40
The Dentists' Menorandum,.................................................................... 50
The People's Dental Journal,.................................................................. 54
The Cincinnati Dental Society,................................................................ 77
Treatment of diseases by oxygen gas,...................................................... 91
Theory and Practice, by W. H. Atkinson,................................................ 125
The Patent Rubber,.................................................................................. 144
The Mississippi Valley Association,........................................................ 191
The use of the mallet, by W. H. Atkinson,............................................... 199
Treatment of fangs,.................................................................................. 215
Treatment of hare-lip,.............................................................................. 230
The American Dental Association,.......................................................... 347
Transactions of the American Dental Association,.................................. 277
To remove amalgams and refill with gold,.............................................. 290
Trip East,................................................................................................. 319
The American Dental Convention,.......................................................... 364
The phosphates in Dental Hygiene,........................................................ 401
The nutrition of the external table of the skull,...................................... 412
Treatment of dental pulp, by George B. Snow,........................................ 425
The moisture of the air,.......................................................................... 458
The mallet,............................................................................................... 462
The People's Journal,.............................................................................. 462
Test for olive oil, ..................................................................................... 502

Western New York Dental Society,.......................................................... 507
What about India Rubber?...................................................................... 519
With us again,.......................................................................................... 546

### OHIO DENTAL COLLEGE ASSOCIATION,

WILL meet in the College building, on Wednesday, the 18th of February, at 9 o'clock, A. M. All the members of the Association are urgently requested to be present, or if it is impossible for some to be present, let them, by all means, empower some one to act for them, as there will be business, before that meeting, intimately connected with their interests, and vastly influencing the interests and welfare of the institution. Almost everything pertaining to the institution requires attention, and it is desirable that every one interested should be present, and contribute his effort.

We hope no one will fail to give prompt attention to this notice. T.

### MISSISSIPPI VALLEY ASSOCIATION.

THIS Association will meet on Tuesday, the 17th of February, at 2 o'clock, P. M., in the lecture room of the Ohio Dental College.

Though last year there was no meeting of this society, we think such will not be the case this year. The same causes for failure do not now exist as then, and we hope to have a good meeting. Business has been good, money is plenty, and the intense excitement, consequent upon the condition of things, has subsided, and men can now think of something else than the war.

We feel confident, from assurances we have received, that we will have a full and good meeting. Let every member be present

T.

### CONTENTS.

Ohio Dental College Association .......... 53

Mississippi Valley Association .......... 53

Revival .......... 54

The People's Dental Journal .......... 54

THE

### DENTAL REGISTER,

OF THE WEST.

Issued Monthly, at $3 a year in advance.

DEVOTED TO THE INTERESTS OF THE DENTAL PROFESSION.

Edited by J. TAFT and GEO. WATT.

The Volume commences in January and closes in December.

The Register being issued monthly is always fresh, therefore indispensable to the practitioner who desires to keep posted up with the new inventions and improvements which are daily developed. Neither expense nor effort will be spared to give value and variety to its contents. Articles will be illustrated with well executed engravings, whenever they will add to the interest or assist in explanation.

Its extensive circulation and frequency of issue makes it the BEST DENTAL ADVERTISING MEDIUM in the United States.

☞CONTRIBUTIONS SOLICITED.

Address Original Communications to Geo. Watt, Xenia, O.

Exchanges to J. Taft, or Dental Register, Cincinnati, Ohio.

Business Communications to

J. TAFT, Publisher,
56 West Fourth Street, Cincinnati, O.

JNO. T. TOLAND, Proprietor,
38 West Fourth Street, Cincinnati, O.

Communications must reach the Editor as early as the 15th, to insure insertion in the next issue.

